# Effect of Sulforaphane on NOD2 via NF-κB: implications for Crohn’s disease

**DOI:** 10.1186/s12950-015-0051-x

**Published:** 2015-01-20

**Authors:** Danielle L Folkard, Gareth Marlow, Richard F Mithen, Lynnette R Ferguson

**Affiliations:** Food and Health Programme, Institute of Food Research, Norwich Research Park, Norwich, UK; Nutrigenomics New Zealand, University of Auckland, Private Bag 92019, Auckland, 1142 New Zealand

**Keywords:** Sulforaphane, NOD2, Inflammation, Crohn’s disease

## Abstract

**Background:**

Sulforaphane has well established anti-cancer properties and more recently anti-inflammatory properties have also been determined. Sulforaphane has been shown to inhibit PRR-mediated pro-inflammatory signalling by either directly targeting the receptor or their downstream signalling molecules such as the transcription factor, NF-κB. These results raise the possibility that PRR-mediated inflammation could be suppressed by specific dietary bioactives. We examined whether sulforaphane could suppress NF-κB via the NOD2 pathway.

**Methods:**

Human embryonic kidney 293T (HEK293T) cells were stably transfected with NOD2 variants and the NF-κB reporter, pNifty2-SEAP. The cells were co-treated with sulforaphane and MDP and secreted alkaline phosphatase (SEAP) production was determined.

**Results:**

We found that sulforaphane was able to significantly suppress the ligand-induced NF-κB activity at physiologically relevant concentrations, achievable via the consumption of broccoli within the diet.

**Conclusions:**

These results demonstrate that the anti-inflammatory role of sulforaphane is not restricted to LPS-induced inflammatory signalling. These data add to the growing evidence that PRR activation can be inhibited by specific phytochemicals and thus suggests that diet could be a way of controlling inflammation. This is particularly important for a disease like Crohn’s disease where diet can play a key role in relieving or exacerbating symptoms.

## Background

### Nucleotide-binding oligomerization domain containing protein 2

Nucleotide-binding oligomerization domain containing protein 2 (NOD2) was first identified in 2001 [1]. NOD2 is composed of two N-terminal caspase recruitment domains (CARDs), a central NOD domain and C-terminal Leucine rich repeats (LRRs). The NOD domain behaves to facilitate self-oligomerization following binding of its ligand and also demonstrates ATPase activity. The LRR domain is important in ligand recognition and the CARDs allow interactions with downstream effecter molecules, ultimately resulting in the recruitment and activation of caspases and the activation of NF-κB [2].

NOD2 functions as an intracellular Pattern Recognition Receptor (PRR) for muramyl dipeptide (MDP) [3]. MDP is a constituent of peptidoglycan (PGN), a component found in the cell walls of both Gram-positive and Gram-negative bacteria and as such NOD2 functions as a sensor for most if not all bacteria [2]. NOD2 has a role in the innate immune response, it identifies Pathogen Associated Molecular Patterns (PAMPs) and stimulates NF-κB [4].

The NOD2 protein is unlike other pattern-recognition receptors such as the Toll-like receptors (TLRs) in that they are not expressed on the surface of the cell but instead are found mainly in the cytosol [5].

The current model of NOD2-mediated NF-κB activation is that LRR’s recognize MDP causing a conformational change allowing NOD2 oligomerization through the NBD. This oligomerization induces RICK (also known as RIP2) which interacts with IKKγ (also known as NEMO) which recruits the TAK1 complex resulting in the activation of the IKK complex, through phosphorylation of IKKβ subunit. This is followed by the degradation of IκBα, which releases NF-κB to activate target genes. MDP recognition by NOD2 also results in the activation of mitogen-activated protein kinases (MAPKs) [6].

Homozygous mutations in NOD2 account for 10 – 15% of patients with Crohn’s disease (CD) [7,8]. To date there has been more than 60 reported mutations in the NOD2 gene, 80% of which have been associated with CD [9]. There are three common single nucleotide polymorphisms (SNPs), that are located in or near the Leucine-rich repeat (LRR) region of NOD2, Arg702Trp (R702W), Gly908Arg (G908R) and a frameshift insertion at L1007fs (Leu1007fsinsC also known as 3020insC) which introduces a premature stop codon [9,10]. Since these mutations are located within or near the C-terminal LRR domain [2,7], they reduce efficient binding of MDP. The frameshift mutation was found to completely abolish the induction of NF-κB activity, while the amino acid substitution SNPs R702W and G908R retained a lower level of induction [11-13]. These results were found to be repeated in both transfected cell lines and in PBMCs from CD patients with the frameshift mutation in NOD2 [3,12].

### Sulforaphane

Broccoli, a member of the cruciferous family of vegetables has long been held to have beneficial health properties, due to its high content of phytochemicals, specifically the glucosinolate, glucoraphanin. Glucoraphanin is hydrolysed by myrosinase to the biologically active isothiocyanate, sulforaphane (SFN). The highest levels of glucoraphanin are found in broccoli seeds, containing 20 – 50 X [14,15] more than mature broccoli. A new broccoli species is now on the market, Beneforté broccoli, that has been bred to contain 2–3 times the level of glucoraphanin as other commercial broccoli [16].

Diets rich in cruciferous vegetables and hence SFN have well established anti-cancer properties for numerous cancers, including prostate, breast, colorectal, lung [17]. More recently it has been shown that it also has important anti-inflammatory properties, including reducing cardiovascular disease mortality [18] and effects against other pathologies including damage to the brain, kidney, liver and heart [19]. With particular relevance to inflammation, SFN has been shown to bind to the LPS receptor, toll-like receptor-4 (TLR4) in addition to blocking the activation of its downstream transcription factor, NF-κB [20]. NF-κB is responsible for controlling the expression of a wide range of pro-inflammatory cytokines in response to inflammatory signals, of which LPS is an example [20].

Studies have shown that phytochemicals, such as SFN, can inhibit PRR-mediated pro-inflammation by either directly targeting the receptor or their downstream signalling molecules [21]. These results raise the possibility that PRR-mediated inflammation could be suppressed by a specific dietary intervention.

In this study we examined if SFN could suppress NF-κB via the NOD2 pathway. We also examined the difference between two types of SFN that are commercially available, the racemic D, L- and the L- isomer. While it has been hypothesised that only the L-isomer is bioactive, the mixture is more representative of what is produced via broccoli consumption.

## Methods

### Reagents

L and D, L-SFN (98% purity) were supplied from LKT laboratories. A stock solution of 25 mg/ml (141 mM) was prepared in DMSO. Muramyl dipeptide (MDP) was supplied from Invivogen. A stock solution was prepared in sterile endotoxin-free water at a concentration of 5 mg/ml. The cell proliferation reagent 4-[3-(4-Iodophenyl)-2-(4-nitrophenyl)-2H-5-tetrazolio]-1,3-benzene disulfonate (WST-1) was purchased from Roche Applied Science. QUANTI-Blue™, used for the detection of secreted embryonic alkaline phosphatase (SEAP) was purchased from Invivogen.

### Cell culture

Human embryonic kidney 293T (HEK293T) cells were chosen for this assay as they express no or very little endogenous TLR4 and do not respond to LPS [22], therefore will allow us to determine the effect of SFN on NOD2 pathway without any crosstalk from TLR4. HEK293T cells were stably transfected with NOD2 variants, either NOD2 WT (pUNO-hNOD2a) or the G908R SNP, and the pNifty2-SEAP plasmid (NF-κB reporter). Expression of NOD2 was confirmed by immunoblotting. Cells were maintained in Dulbecco’s Modified Eagle Media (DMEM) supplemented with 10% fetal bovine serum (Invitrogen, NZ), 1% penicillin/streptomycin, 0.06% blasticidin (Invivogen, NZ) and 0.1% Zeocin™ (Invitrogen, NZ) at 37°C, 5% CO_2_.

### NF-κB activity affected by SFN

The stably transfected HEK293T were re-suspended in antibiotic-free media and seeded at a density of 1 × 10^5^ cells/ml for NOD2 WT and 1 × 10^4^ cells/ml for NOD2 G908R. Cells were cultured for 48 hours before being co-treated with SFN at a range of concentrations (1 – 200 μM) to determine an optimal dose (0.5 – 10 μM) and a range of MDP (Invivogen) concentrations (0.2 – 4 μg/ml) or media equivalent. After a further 24 hours SEAP production was measured at 650 nm, using QUANTI-Blue™ and cell viability was determined using WST-1 read at 450 nm. Activity was normalised and scored in relation to control. Toxicity was scored based on dilutions resulting in <70% of control.

### Statistical analysis

All tests were carried out on at least three independent experiments, and performed in duplicate for each of the treatments. IC_50_ values were compared using the Student’s *T*-test. Statistical analysis was performed by one way ANOVA followed by the Bonferroni multiple comparison test.

## Results

**Figure 1:**
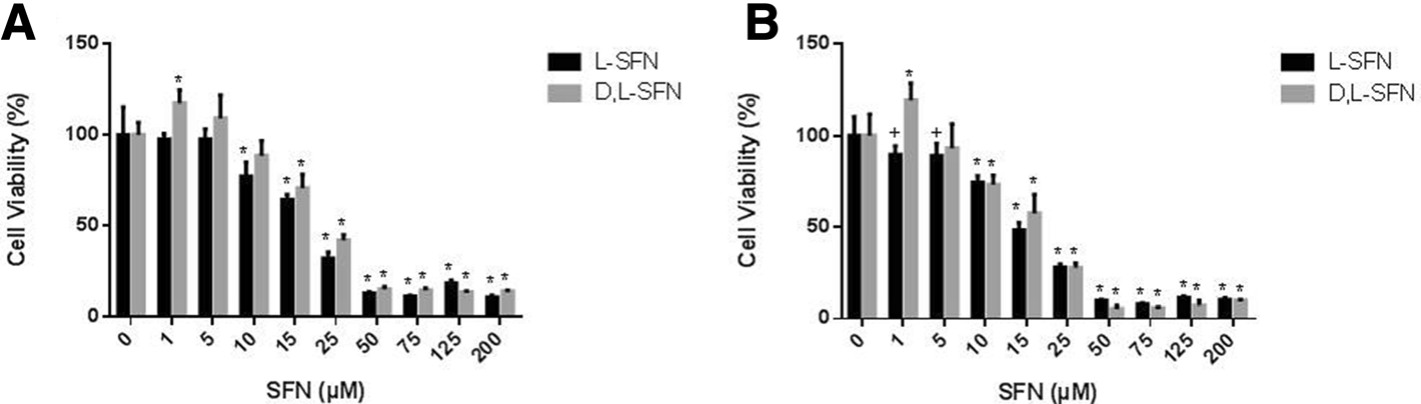
**Effect of SFN on viability of NOD2 WT (A) and NOD2 G908R (B) cells.** Cells were treated with L- or D, L-SFN for 24 h at concentrations ranging from 0–200 μM with DMSO as a control. Data shown = mean ± SD. *p ≤ 0.001, +p ≤ 0.01 as compared to cells only.

Initially we examined if SFN has any effect on cell viability in both NOD2 WT and NOD2 G908R cells (Figure 1). IC_50’s_ were subsequently determined for both isoforms of SFN in the NOD2 WT and NOD2 G908R cell lines. There was no significant difference in the effect of either L- or D, L- SFN in NOD2 WT cells, with an IC_50_ of 18 μM. However the IC_50’s_ were significantly lower in the NOD2 G908R cells, L-SFN IC_50_ was calculated at 17 μM which was significantly higher than for the D, L-isoform (13 μM) (Table 1). Based on these results we selected a dose range of SFN up to 10 μM.

**Table 1:** **Response to the different isomers of SFN in NOD2 WT and NOD2 G908R cells**

**Cells**	**SFN**	**IC**_**50**_
NOD2 WT	L	18.84 (1.09)
NOD2 WT	D, L	18.03 (1.92)
NOD2 G908R	L	16.9 (0.82)
NOD2 G908R	D, L	12.93 (1.19)

Mean ± SD, statistically analysed using Student’s *T*-test.

**Figure 2:**
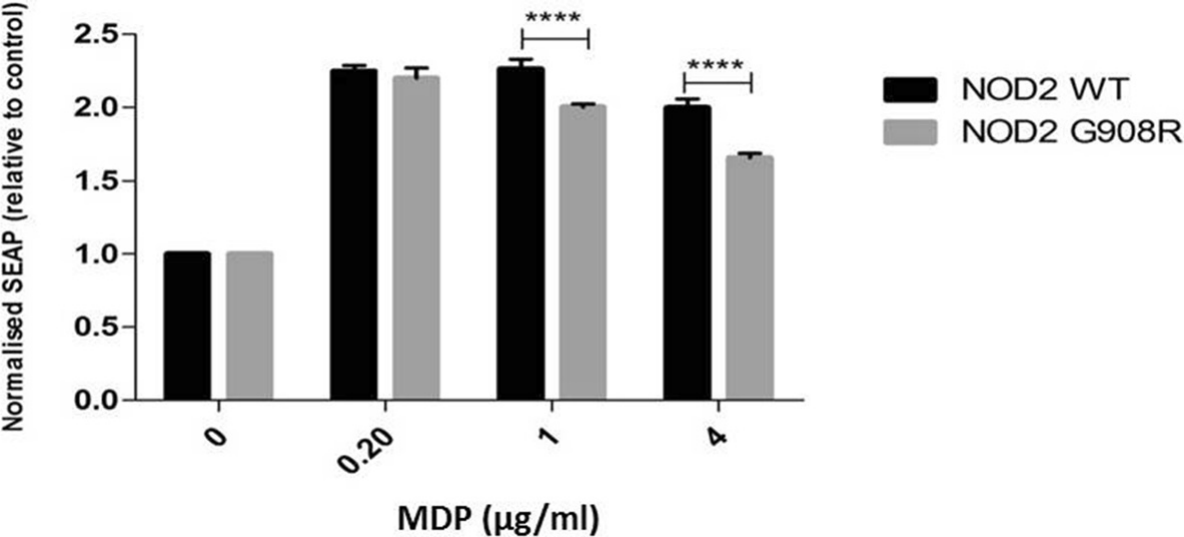
**Cells expressing G908R NOD2 receptor are less responsive to ligand activation.** Cells were treated with MDP (0.2-4 μg/ml) with endotoxin-free water as a control for 24 h. After 24 h, the media was collected and added to QUANTI-Blue™ solution for analysis of the SEAP. SEAP levels were measured via a spectrophotometer at 650 nm. Data shown = mean ± SEM, n = 4. ****p < 0.0001.

Secondly we confirmed the appropriate MDP dose to use to stimulate the cells. Our results showed that all tested doses significantly induced the activity of the reporter gene in both the wildtype and the variant G908R NOD2 cells. The WT cells demonstrated significantly higher levels of induction compared to the variant cells, as expected at 1 and 4 μg/ml (Figure 2). We observed no significant toxicity at any dose of MDP tested.

**Figure 3:**
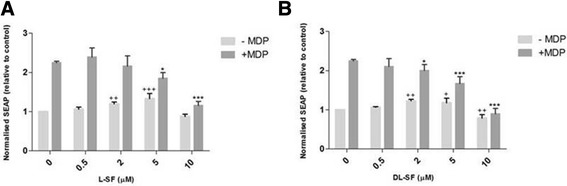
**L- and D, L-SFN are able to significantly suppress MDP-induced SEAP activity in NOD2 WT cells.** NOD2 WT cells were treated for 24 h with 0.2 μg/ml MDP in the presence or absence of L- **(A)** or D, L-SFN **(B)**. Media was collected and added to QUANTI-Blue™ solution and left at room temperature for 40–60 min before being measured using a spectrophotometer at 650 nm. Data shown = mean ± SD, data representative of three independent experiments. + p < 0.05, ++ p < 0.01 comparison to 0 μM –MDP. *p < 0.05, **p < 0.01, ***p < 0.001 comparison to 0 μM + MDP.

With the assay conditions optimised we next measured the effect of SFN at physiologically appropriate concentrations on ligand induction in NOD2 WT cells (Figure 3). At all three concentrations of MDP, both the L- and D, L-SFN isoforms significantly suppress the ligand-induced NF-κB activity as measured through the levels of secreted alkaline phosphatase (SEAP) within the supernatant at 5 and 10 μM SFN.

**Figure 4:**
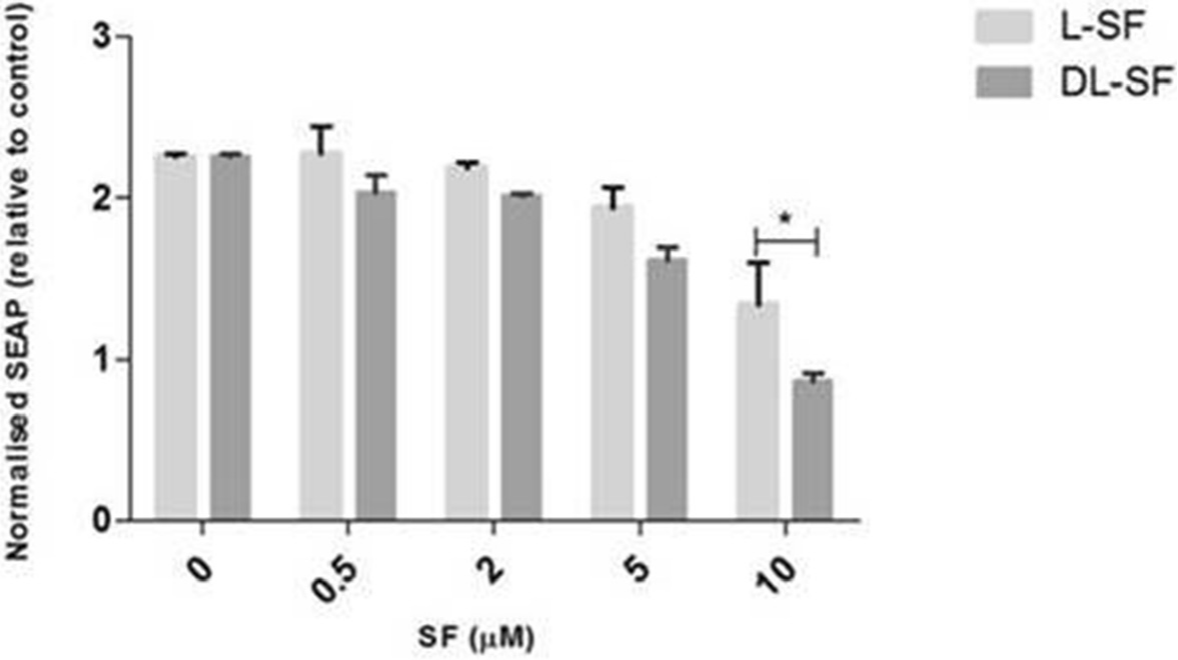
**L- and D, L-SFN do not show consistent significant differences between their effects on MDP-induced NF-** **κ** **B activity.** NOD2 WT cells were treated for 24 h with 0.2 μg/ml MDP in the presence or absence of L- or D, L-SFN (0.5, 2, 5 or 10 μM). Media was collected and added to QUANTI-Blue™ solution and left at room temperature for 40–60 min before being measured using a spectrophotometer at 650 nm. Data shown = mean ± SEM, n = 3. *p < 0.05.

**Figure 5:**
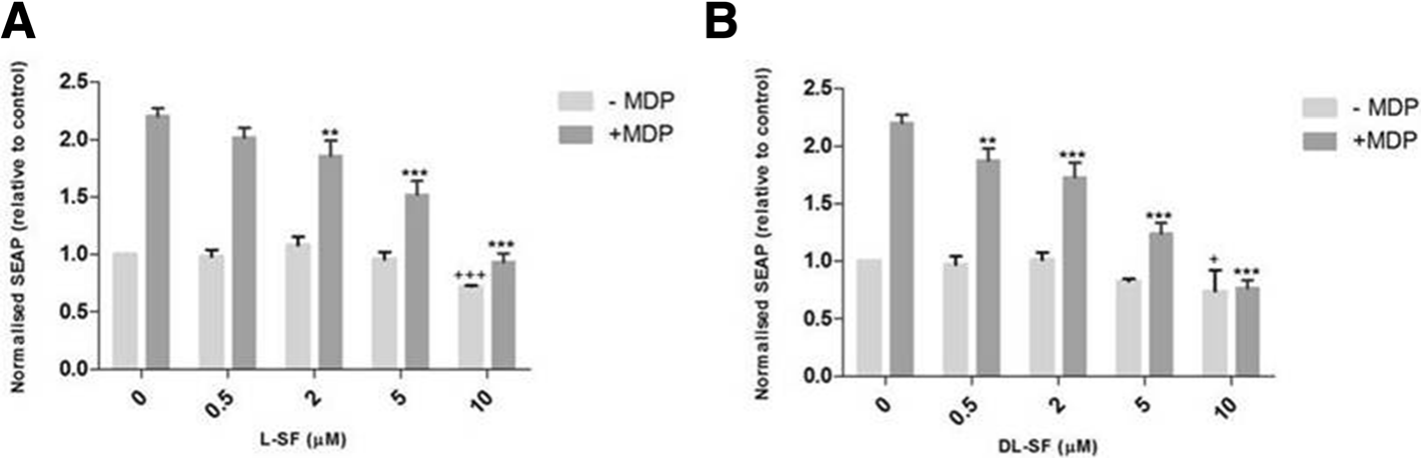
**L- and D, L-SFN are able to significantly suppress MDP-induced SEAP activity in NOD2 G908R cells.** NOD2 G908R cells were treated for 24 h with 0.2 μg/ml MDP in the presence or absence of L- **(A)** or D, L-SFN **(B)**. Media was collected and added to QUANTI-Blue™ solution and left at room temperature for 5-15 min before being measured using a spectrophotometer at 650 nm. Data shown = mean ± SD, data representative of three independent experiments. + p < 0.05, ++ p < 0.01, +++ p < 0.001 comparison to 0 μM –MDP. *p < 0.05, **p < 0.01, ***p < 0.001, comparison to 0 μM + MDP.

**Figure 6:**
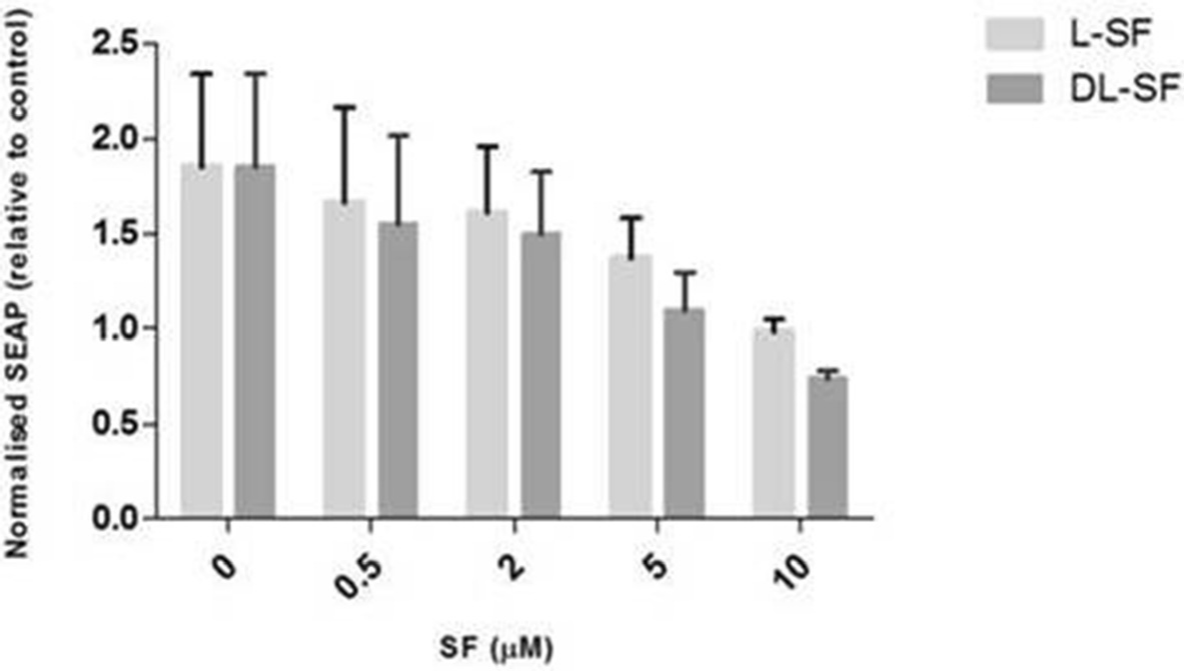
**L- and D, L-SFN do not show any significant differences between their effects on MDP-induced NF-**κ**B activity in NOD2 G908R cells.** NOD2 G908R cells were treated for 24 h with 0.2 μg/ml MDP in the presence or absence of L- or D, L-SFN (0.5, 2, 5 or 10 μM). Media was collected and added to QUANTI-Blue™ solution and left at room temperature for 5-15 min before being measured using a spectrophotometer at 650 nm. Data shown = mean ± SEM, n = 3.

We subsequently confirmed if there was a differential effect based on SFN isomer in the NOD2 WT cells (Figure 4). The only significant difference was seen at 10 μM SFN stimulated with 0.2 μg/ml MDP (Figure 4). This was not as a result of reduced viability with D, L-SFN (data not shown). Finally we examined if the same suppression was true in NOD2 G908R cells. Similarly to the NOD2 WT cells, both SFN isomers showed significant reductions at nearly all the concentrations used (Figure 5). No significant differences were found between the suppression of MDP-induced NF-κB activity when using the L- or the D, L-SFN forms (Figure 6).

## Discussion

We observed a significant 2-fold response to MDP in NOD2 WT cells, similar to that previously described by our group [23] in the original assay development. It was expected that NOD2 G908R cells, would have a lower response to MDP than NOD2 WT cells and this was shown to be the case, with a significant reduction in response of NOD2 G908R at MDP concentrations of 1 and 4 μg/ml. However the reduction in response was not as large as has previously been reported by Bonen [11] or Inohara [12]. The response we observed was more similar with the most recent study by Lecine [13]. However these studies all use different concentrations of MDP and different methods to determine effects which may account for the differences observed.

We found that SFN was able to significantly suppress the ligand-induced NF-κB activity at concentrations of 5 and 10 μM in both NOD2 WT and NOD2 G908R cells. Surprisingly, given the hypothesis that only the L-isoform of SFN is bioactive, we found no significant difference between the effects of the two isoforms at the concentrations tested.

In both the NOD2 WT and the variant NOD2 G908R cells, SFN is able to significantly suppress MDP-induced NF-κB activity at physiologically relevant concentrations, achievable via the consumption of broccoli within the diet. These results demonstrate that the anti-inflammatory role of SFN is not restricted to only LPS-induced inflammatory signalling. As the anti-inflammatory effect is seen in both the WT and G908R NOD2 variant it appears that SFN is not directly targeting the NOD2 receptor but either downstream signalling molecules or possibly dimerization of the NOD2 molecule itself similar to that observed with SFN inhibited TLR4-mediated NF-κB activation [21]. A possibility is that SFN is targeting a common component of both the NOD2 and TLR4 pathway signalling pathways, such as TAK1, the convergent point of the MDP and LPS signalling pathways [24,25]. It has been observed that NOD2 ligands can regulate or amplify the immune response to other pathogenic components including TLR4 [24,26]. It has also been implied that MDP-triggered mechanisms are involved in late stage TLR4-mediated events [25]. This may provide a further possible explanation of how SFN is having an effect on both NOD2 and TLR4, but requires further study to confirm.

The fact that SFN affects both WT and the G908R variant is encouraging for CD patients, as stated earlier there are over 60 reported NOD2 variants, of these 80% have been associated with CD [9]. There is still debate as to whether NOD2 mutations are gain or loss of function in CD [27,28]. As SFN doesn’t directly target NOD2 it may be beneficial for all of these cases. This means if CD patients can tolerate foods containing SFN they may benefit from its anti-inflammatory properties and thus have reduced flare-ups and alleviate other symptoms.

## Conclusion

These results add to the growing evidence that PRR activation can be inhibited by specific phytochemicals and thus suggest that dietary components could be a way of controlling inflammation. This is particularly important in a disease like CD where diet can play a key role in relieving or exacerbating symptoms [29]. Unfortunately broccoli, one of the main sources of SFN has shown mixed results in food tolerance questionnaires of people with CD with 20% saying it makes symptoms worse [29]. However, there are supplements available which provide the levels of SFN required without the associated side effects of eating broccoli. Supplementation with SFN could result in a reduction in pro-inflammatory signalling in CD patients and hence alleviate some of the associated symptoms.

The next step would be to further characterise the mechanism of action of SFN and to identify where it interacts within the NOD2 pathway and if this would be beneficial to CD patients. This could be achieved by analysing expression levels of genes and proteins associated with NOD2 and CD. Ideally we would like to be able to conduct a dietary intervention trial, using normal and glucoraphanin enhanced Beneforté broccoli, which would show us the direct effect of SFN and not any other phytochemical within broccoli.
